# A Single-center Experience: Does MRI-guided Target Prostate Biopsy Meet Expectations?

**DOI:** 10.7759/cureus.6160

**Published:** 2019-11-14

**Authors:** Türev Demirtaş, Gökhan Sönmez, Şevket T Tombul, Abdullah Demirtaş

**Affiliations:** 1 History of Medicine and Ethics, Erciyes University, Kayseri, TUR; 2 Urology, Kayseri City Hospital, Kayseri, TUR; 3 Urology, Erciyes University, Kayseri, TUR

**Keywords:** biopsy, combined, target, prostate, fusion

## Abstract

Objective

Target biopsy (TB) was defined to detect a higher rate of cancer with fewer cores. Today, however, the combined biopsy (CB; TB + standard prostate biopsy (SPB)) with even more cores has become more popular. We aimed to compare CB results with those of TB and SPB in patients in the gray zone and, based on the outcomes, to determine whether TB has achieved its goal based on the expectation that higher cancer detection rates can be attained with fewer cores.

Materials and methods

This prospective study included patients with a prostate imaging reporting and data system (PI-RADS) ≥3 lesion and serum prostate-specific antigen (PSA) <10 ng/ml who underwent CB. All patients underwent two to five core biopsies per suspicious lesion (TB). Then, an SPB was administered to the same patients and in the same sessions. For fusion biopsy procedures, a fusion ultrasonography device with rigid software was used.

Results

A total of 404 patients were included in the study. The rate of clinically significant prostate cancer (sPCa) detection in TB, SPB, and CB was 30.2%, 25.5%, and 38.4%, respectively (p<0.05). The highest sPCa detection rate per core was detected in TB. For these patients, the CB results were accepted as the reference standard and then the histopathological upgrading of the lesions detected by SPB and TB was determined. Accordingly, higher histopathological upgrade rates were detected in SPB (10% and 25.7%).

Conclusion

We can say that the philosophy of detecting more cancers with a low number of cores, which was created when defining TB, was partially unsuccessful.

## Introduction

Prostate cancer (PCa) is the second most common cancer in men worldwide [1]. Digital rectal examination (DRE), serum prostate-specific antigen (PSA) test, and prostate needle biopsy are the most common procedures used in the diagnosis of PCa [2].

Standard prostate biopsy (SPB) remains the golden standard in the diagnosis of PCa. However, it has been shown to result in a missed diagnosis of PCa in 21%-28% and to detect lower-grade tumors in 14%-17% of the cases [3]. Accordingly, a number of biopsy techniques have been developed in line with advancing technology. Of these, multiparametric magnetic resonance imaging (mpMRI)-guided fusion prostate biopsy (FPB) has recently emerged as a promising technique, which is based on the prostate imaging reporting and data system (PI-RADS) scoring system and is performed in the form of cognitive, fusion, or in-bore biopsy using ultrasound fusion imaging with a computer program [4].

FPB is recommended primarily for repeat biopsy in patients with a clinical suspicion of PCa whose initial prostate biopsies were negative and for follow-up biopsy in PCa patients on active follow-up and for patients who have a clinical suspicion of recurrence after local minimally invasive treatment (e.g., radiotherapy or high-intensity focused ultrasound (HIFU)) [5-6]. Additionally, FPB has been recently shown to provide effective outcomes in biopsy-naïve patients [7-9].

MpMRI-targeted biopsy (TB), a part of fusion biopsy, was first introduced in the early 2010s, predominantly to investigate whether better outcomes can be obtained by performing a biopsy on target lesions with fewer cores [10-11]. In later years, however, combined prostate biopsy (CB) (SPB + TB), which adopted a higher number of cores and was shown to have higher cancer detection rates, became a popular technique [7,12-13].

In the present study, we aimed to compare CB results with those of TB and SPB in patients with a PI-RADS ≥3 lesion and PSA <10 ng/ml and, based on the outcomes, to determine whether TB has achieved its goal based on the expectation that higher cancer detection rates can be attained with fewer cores.

## Materials and methods

Patient selection and pre-biopsy procedure

The prospective study included patients that were followed up at Erciyes University Urology Clinic with a clinical suspicion of PCa and a PSA level of <10 ng/ml. Patients detected with no suspicious lesions on mpMRI prior to the biopsy procedure and patients with a PI-RADS <3 lesion were excluded from the study. Clinical and demographic characteristics, including age, body mass index (BMI), serum PSA level, total prostate volume, PI-RADS score, the number of biopsy cores used in the procedure, and histopathological diagnosis were recorded for each patient. Prior to biopsy, all the patients had sterile urine culture and the ongoing anticoagulant and antiaggregant therapies of the patients were discontinued. Twenty-four hours prior to the procedure, three oral doses of 750 mg of ciprofloxacin (at a 12-h interval) were administered. No bowel preparation was used prior to the procedure. Patients with a histopathological diagnosis of atypical small acinar proliferation (ASAP) or high-grade prostatic intraepithelial neoplasia (HGPIN) were excluded from the study and were referred for appropriate treatment and follow-up.

MpMRI and PI-RADS

Prior to the biopsy procedure, prostate MpMRI was performed without an endorectal coil in each patient using a Siemens Magnetom 1.5 Tesla MRI device (Siemens Medical Solutions, Malvern, USA). Suspicious lesions detected on contrast-enhanced T1, T2, and diffusion-weighted sequences were evaluated based on the PI-RADS version 2 [14]. In patients with multiple lesions and more than one PI-RADS score, only the highest PI-RADS score was considered.

Standard prostate biopsy and fusion prostate biopsy

The entire biopsy procedure was performed in polyclinic conditions with local or sedation anesthesia. Transrectal ultrasonography (TRUS) was performed by using an ultrasonography (US) system with rigid fusion software (LOGIQ E9; General Electric, MA, USA) with the patients lying in the left decubitus position. Rectal lidocaine gel was applied before a rectal US probe was introduced. A sonographic examination of the prostate tissue was performed to determine the presence of prominent lesions. The total prostate volume was measured using the following formula: height (H) x width (W) x length (L) x 0.523. MpMRI images were transferred to the US system. Following the segmentation (matching) of sonographic images with MRI images, the lesions detected on MpMRI were marked. Subsequently, the periprostatic block was administered with 2% prilocaine hydrochloride (20 mg/mL) injected into the neurovascular bundle on both sides of the prostate, with 5 mL to the right and 5 mL to the left. Following the block, two to five core biopsies were obtained from the MRI-targeted lesions with PI-RADS ≥3. All the data transfers and markings throughout the procedure were performed by two urologists experienced and trained in transrectal prostate ultrasonography and biopsy. Following the completion of TB, a standard 12-core SPB was performed in each patient after impairing MpMRI segmentation.

Histopathological examination

The specimens were separately placed in previously labeled containers and were sent for histopathological examination. The cancer detection rate per core was determined in accordance with the International Society of Urological Pathology (ISUP) grades that are calculated based on primary and secondary Gleason scores [15]. Clinically significant prostate cancer (sPCa) was considered as biopsy Gleason score ≥3+4 or maximum cancer core length ≥5 mm.

Statistical analysis

Data were analyzed using SPSS for Windows version 22.0 (Armonk, NY: IBM Corp.). The normal distribution of data was determined using the Shapiro-Wilk test. Continuous variables with normal distribution were expressed as mean ± standard deviation (SD) and the continuous variables with non-normal distribution were expressed as median (first to third quartile). Categorical variables were expressed as percentages. Categorical dependent variables were compared using McNemar’s test and Cochran's Q test. Non-normally distributed continuous dependent variables were evaluated using Friedman’s test. A p-value of <0.05 was considered significant.

## Results

Of the 778 patients that underwent mpMRI within the study period, a total of 404 patients with a PI-RADS ≥3 lesion and PSA<10 ng/ml who underwent CB were included in the study. The mean age was 62.38±7.19 years, the mean BMI was 27.57±3.76 kg/m^2^, and the median PSA was 7.50 (5.40-9.34) ng/ml. A biopsy procedure was performed under local anesthesia in 217 (53.7%) patients. A PI-RADS 3 lesion was the most common lesion detected in the patients (58.9%) and ISUP grade 1 was the most common histopathological grade detected in our patients (59.4%). Of the 404 patients, 130 (32.2%) patients had a history of negative biopsy and 274 (67.8%) patients were biopsy naïve. Table 1 presents some demographic and clinical data of patients. The histopathological analysis of SPB, TB, and CB indicated that CB provided the highest sPCa detection rate (38.4%) and TB provided the lowest rate (25.5%), and a significant difference was found among the three groups (p<0.001). In contrast, the highest cancer detection rate per core was detected in the TB group (Table 2). The total number of patients detected with sPCa by all three biopsy methods was 70 (17.3%). For these patients, the CB results were accepted as the reference standard and then histopathological upgrading of the lesions detected by SPB and TB was determined (Table 3). Accordingly, histopathological upgrading was detected in 18 (25.7%) and 7 (10%) of the lesions detected by SPB and TB, respectively (p=0.043).

**Table 1:** Demographic and clinical data of all patients included in the study PSA: Prostate-specific antigen; sPCa: Clinically significant prostate cancer; PI-RADS: Prostate Imaging-Reporting and Data System; ISUP: International Society of Urological Pathology

Parameter	Value
Age (years)	62.38 + 7.19
Body Mass Index (kg/m^2^)	27.57 + 3.76
Total PSA (ng/ml)	7.50 (5.40-9.34)
Prostate Volume (mm^3^)	56.01 (42.21-79.90)
Types of Anesthesia Sedation Local	187, 46% 217, 53%
PI-RADS/sPCa rates: 3; 4; 5	64/238, 26.9%; 54/104, 51.9%; 37/62, 59.7%
sPCa for Biopsy Naives	111/274 (40.5%)
sPCa for Seconder Patients	44/130 (33.8%)
Overall sPCa Rates	155/404 (38.4%)
ISUP Scores (n, %): 1; 2; 3; 4; 5	92, 59.4%; 25, 16.1%; 15, 10.0%; 17, 11.0%; 6, 1.5%

**Table 2:** Comparison of histopathological results according to biopsy type SPB: Standard prostate biopsy; TB: Targeted biopsy; CB: Combined biopsy

	SPB^a^	TB^b^	CB^c^	p
Number of cores, median	12.0 (12.0-12.0)	4.0 (4.0-6.0)	16.0 (16.0-18.0)	<0.001
Clinically significant prostate cancer	103/404 (25.5%)	122/404 (30.2%)	155/404 (38.4%)	P^ab^: 0.035 P^ac^: <0.001 P^bc^: <0.001
Cancer detection rate per core	668/4848 (13.8%)	386/1885 (20.5%)	1054/6733 (15.7%)	<0.001

**Table 3:** Histopathological upgrade rates of standard biopsy and target biopsy results of 70 patients with malignancy detected by all biopsy methods (histopathological results of combined biopsy were accepted as the reference result)

	Type of Biopsy (n=70)			
	Standard Biopsy^a^	Target Biopsy^b^	Overall	P^ab^
Upgrade rates	18/70 (25.7%)	7/70 (10%)	25/70 (35.7%)	0.043

## Discussion

MpMRI-guided FPB has been shown to provide successful outcomes in patients with a history of negative biopsy and has become a well-established biopsy technique [5-6]. Additionally, it has recently emerged as a successful technique in biopsy-naïve patients as well [7-9]. As expected, based on these data, the findings of the present study that evaluated both biopsy-naïve and previous negative biopsy patients indicated that TB had the lowest number of core and CB had the highest (p<0.001). However, TB could not show the same success in the cancer detection rate as it had the lowest rate when compared to SPB and CB (25.5%, 30.2%, and 38.4%, respectively).

The finding of the present study that suggested that CB had the highest cancer detection rate was consistent with the literature. Nevertheless, the finding that suggested that TB had the lowest detection rate contradicted with the literature. A recent systematic review indicated that SPB had a cancer detection rate of 26.3%-56.6% while CB had a rate of 33.7%-79.5% [16]. Another study compared the three techniques and revealed that CB had an sPCa detection rate of 43% [13]. The study was dissimilar to our study in that it reported that the sPCa detection rates were 31% and 36% in the TB and SPB groups, respectively (p=0.155). Another study revealed that CB had an sPCa detection rate of 65% [12]. The same study, unlike our study, did not only include patients with PSA<10 ng/ml but included patients with all PSA levels and reported that the patients had a median PSA level of 9 (range, 0.7-48) ng/ml, both of which might be the reasons for the higher cancer detection rates determined in that study as compared to those of our study. Moreover, in the same study, the sPCa detection rates for TB and SPB were 38.2% and 33.5% and no significant difference was found between the two rates (p=0.2). In another recent study, Baco et al. evaluated biopsy-naïve patients and revealed similar sPCa detection rates for two-core TB and 12-core random biopsy, both of which were administered only in patients with suspicious lesions (44% and 49%, respectively) (p=0.5) [17]. In our study, however, TB provided poorer outcomes as compared to SPB. This finding contradicted the literature and could be attributed to several factors, including the learning curve of TB, the inclusion of both primary and secondary patients, and the patient selection criteria of our study.

A previous study evaluated patients with a history of negative biopsy and found an sPCa detection rate of 34.9% for MRI-guided FPB [18]. In a study published in 2015, Borkowetz et al. evaluated biopsy-naïve and previous negative biopsy patients who underwent CB and reported that the cancer detection rate of CB was 44% and 46% in biopsy-naïve and previous negative biopsy patients, respectively [19]. Another study that was conducted by the same authors evaluated biopsy-naïve patients and reported an sPCa detection rate of 44% [20]. In line with the literature, our findings indicated that sPCa was detected in 40.5% and 33.8% of biopsy-naïve and previous negative biopsy patients, respectively.

In our study, the PI-RADS 3 lesion was the most common lesion detected in the patients (58.9%) and the sPCa detection rates in PI-RADS 3, 4, and 5 lesions were 26.9%, 51.9%, and 59.7%, respectively. In a recent study, Boesen et al. reported that a PI-RADS 4 lesion was the most common lesion detected in the patients (49.5%) and the cancer detection rates for PI-RADS 3, 4, and 5 lesions were 22.2%, 62.7%, and 94.1%, respectively [13]. Although the study found similar cancer detection rates to those of our study, the cancer detection rates for PI-RADS 4 and 5 lesions were remarkably higher than those of our study. This difference could be ascribed to the absence of a limitation for the PSA levels of the patients included in that study and to the higher PSA level in that study compared to that of our study (12.80 (8.9-19.6) ng/ml vs. 7.50 (5.40-9.34) ng/ml. Another recent study, however, reported that the sPCa detection rate was 63% for both PI-RADS 4 and 5 lesions [21]. On the other hand, there are also some other studies that suggest that the sPCa detection rate becomes higher as the PI-RADS grade increases [22-23].

Our results also indicated that ISUP grade 1 was the most common histopathological grade detected in our patients (59.4%). Similarly, a previous study conducted in 2013 evaluated a total of 582 patients and revealed that ISUP grade 1 was the most common histopathological grade [24]. In contrast, a more recent study reported that ISUP grade 2 was the most common histopathological grade detected in the patients [13]. However, the patients included in that study had higher PSA levels compared to those of our patients (12.80 vs. 7.50 ng/ml) and had a higher frequency of PI-RADS 4 lesions compared to that of our patients (49.5% vs. 25.7%). These findings implicate that patients with lower PSA levels are likely to be detected with ISUP grade 1 and 2 tumors.

The cancer detection rate per core is an important parameter in the determination of the ideal biopsy technique. Junker et al. found that the cancer detection rate per core was 10.4% in SPB as opposed to 29.3% in TB [23]. Another study evaluated a total of 175 biopsy-naïve patients and reported that TB, despite being performed with fewer biopsy cores, provided a similar prostate cancer detection to that of SPB [17]. Fourcade et al. presented similar findings and reported that the cancer detection rate per core was 29% in TB and 14.8% in SPB [12]. Our findings were consistent with those reported in the literature and indicated that TB had the highest cancer detection rate per core.

In our study, the CB results were accepted as the reference standard. Accordingly, histopathological upgrading was detected in 18 (25.7%) and seven (10%) of the lesions detected on SPB and TB, respectively. A study conducted in 2018 indicated that tumor upgrading occurred in 16.4% and 31.5% of the patients that underwent TB and SPB [24]. However, another retrospective study revealed these rates as 33% and 44%, respectively [25]. Although these studies have reported different upgrading rates for various biopsy techniques, all the studies converged on the conclusion that TB had lower upgrading rates as compared to other biopsy techniques. The findings of our study were consistent with these findings. However, we detected relatively lower upgrading rates, which could be explained by the fact that histopathological upgrading rates were determined based on the CB results rather than the results obtained by radical prostatectomy, considering that CB results are likely to cause a certain amount of upgrading following radical prostatectomy.

Our study had several key limitations. First, it had a small patient population. Second, upgrading rates were calculated based on the CB results in lieu of the results of radical prostatectomy. Third, patients detected with ASAP and HGPIN were excluded from the study, which might have had a slight effect on the results of the study. Finally, the patients had inadequately short follow-up periods, which prevented the documentation of the long-term outcomes of the patients.

## Conclusions

Among the three biopsy techniques evaluated in the study, CB had the highest cancer detection rate. We believe that although TB was introduced into clinical practice based on the hypothesis that “similar cancer detection rates may be attained with fewer cores,” TB could be asserted to have achieved this goal partially and to have given way to the emergence of CB, which, unlike TB, is based on the hypothesis that “more cores can be employed to attain higher cancer detection rates.” Accordingly, it is tempting to consider that the philosophy of CB runs counter to that of TB and thus the philosophy of TB has become unsuccessful. Further large-scale studies are needed to substantiate our findings.

## References

[1] Bray F, Ferlay J, Soerjomataram I, Siegel RL, Torre LA, Jemal A (2018). Global cancer statistics 2018: GLOBOCAN estimates of incidence and mortality worldwide for 36 cancers in 185 countries. CA Cancer J Clin.

[2] Litwin MS, Tan HJ (2017). The diagnosis and treatment of prostate cancer. A review. JAMA.

[3] Bjurlin MA, Carter HB, Schellhammer P (2013). Optimization of initial prostate biopsy in clinical practice: sampling, labeling and specimen processing. J Urol.

[4] Woo S, Suh CH, Kim SY, Cho JY, Kim SH (2017). Diagnostic performance of prostate imaging reporting and data system version 2 for detection of prostate cancer: a systematic review and diagnostic meta-analysis. Eur Urol.

[5] Rosenkrantz AB, Verma S, Choyke P (2016). Prostate magnetic resonance imaging and magnetic resonance imaging targeted biopsy in patients with a prior negative biopsy: a consensus statement by AUA and SAR. J Urol.

[6] Lai WS, Gordetsky JB, Thomas JV, Nix JW, Rais-Bahrami S (2017). Factors predicting prostate cancer upgrading on magnetic resonance imaging-targeted biopsy in an active surveillance population. Cancer.

[7] Sönmez G, Tombul ŞT, İmamoğlu H, Akgün H, Demirtaş A, Tatlışen A (2019). Multiparametric MRI fusion-guided prostate biopsy in biopsy naive patients: preliminary results from 80 patients. Turk J Urol.

[8] Yarlagadda VK, Lai WS, Gordetsky JB, Porter KK, Nix JW, Thomas JV, Rais-Bahrami S (2018). MRI/US fusion guided prostate biopsy allows for equivalent cancer detection with significantly fewer needle cores in biopsy-naive men. Diagn Interv Radiol.

[9] Abd-Alazeez M, Kirkham A, Ahmed HU (2014). Performance of multiparametric MRI in men at risk of prostate cancer before the first biopsy: a paired validating cohort study using template prostate mapping biopsies. Pros Cancer Prostatic Dis.

[10] Franiel T, Stephan C, Erbersdobler A (2011). Areas suspicious for prostate cancer: MR guided biopsy in patients with at least one transrectal US-guided biopsy with a negative finding-multiparametric MR imaging for detection and biopsy planning. Radiology.

[11] Rastinehad AR, Turkbey B, Salami SS (2014). Improving detection of clinically significant prostate cancer: magnetic resonance imaging/transrectal ultrasound fusion guided prostate biopsy. J Urol.

[12] Fourcade A, Payrard C, Tissot V (2018). The combination of targeted and systematic prostate biopsies is the best protocol for the detection of clinically significant prostate cancer. Scand J Urol.

[13] Boesen L, Nørgaard N, Løgager V, Balslev I, Thomsen HS (2017). A prospective comparison of selective multiparametric magnetic resonance imaging fusion targeted and systematic transrectal ultrasound-guided biopsies for detecting prostate cancer in men undergoing repeated biopsies. Urol Int.

[14] Barentsz JO, Weinreb JC, Verma S (2016). Synopsis of the PI-RADS v2 guidelines for multiparametric prostate magnetic resonance imaging and recommendations for use. Eur Urol.

[15] Epstein JI, Egevad L, Amin MB, Delahunt B, Srigley JR, Humphrey PA, Grading Committee (2016). The 2014 International Society of Urological Pathology (ISUP) consensus conference on Gleason grading of prostatic carcinoma: definition of grading patterns and proposal for a new grading system. Am J Surg Pathol.

[16] Gayet M, van der Aa A, Beerlage HP, Schrier BP, Mulders PF, Wijkstra H (2016). The value of magnetic resonance imaging and ultrasonography (MRI/US)-fusion biopsy platforms in prostate cancer detection. BJU Int.

[17] Baco E, Rud E, Eri LM (2016). A randomized controlled trial to assess and compare the outcomes of two-core prostate biopsy guided by fused magnetic resonance and transrectal ultrasound images and traditional 12-core systematic biopsy. Eur Urol.

[18] Abdi H, Zargar H, Goldenberg SL (2015). Multiparametric magnetic resonance imaging-targeted biopsy for the detection of prostate cancer in patients with prior negative biopsy results. Urol Oncol.

[19] Borkowetz A, Platzek I, Toma M (2015). Comparison of systematic transrectal biopsy to transperineal magnetic resonance imaging/ultrasound-fusion biopsy for the diagnosis of prostate cancer. BJU Int.

[20] Borkowetz A, Hadaschik B, Platzek I (2018). Prospective comparison of transperineal magnetic resonance imaging/ultrasonography fusion biopsy and transrectal systematic biopsy in biopsy-naïve patients. BJU Int.

[21] Del Monte M, Leonardo C, Salvo V (2018). MRI/US fusion-guided biopsy: performing exclusively targeted biopsies for the early detection of prostate cancer. Radiol Med.

[22] Grey AD, Chana MS, Popert R, Wolfe K, Liyanage SH, Acher PL (2015). Diagnostic accuracy of magnetic resonance imaging (MRI) prostate imaging reporting and data system (PI-RADS) scoring in a transperineal prostate biopsy setting. BJU Int.

[23] Junker D, Schäfer G, Heidegger I (2015). Multiparametric magnetic resonance imaging/transrectal ultrasound fusion targeted biopsy of the prostate: preliminary results of a prospective single-centre study. Urol Int.

[24] Kayano PP, Carneiro A, Castilho TML (2018). Comparison of Gleason upgrading rates in transrectal ultrasound systematic random biopsies versus US-MRI fusion biopsies for prostate cancer. Int Braz J Urol.

[25] Borkowetz A, Platzek I, Toma M (2016). Direct comparison of multiparametric magnetic resonance imaging (MRI) results with final histopathology in patients with proven prostate cancer in MRI/ultrasonography-fusion biopsy. BJU Int.

